# Editors’ Pick: Contamination has always been the issue!

**DOI:** 10.1186/s13323-014-0017-4

**Published:** 2014-12-30

**Authors:** Antti Sajantila

**Affiliations:** University of Helsinki, Department of Forensic Medicine, P.O.Box 40, 00014 Helsinki, Finland

In the middle of the 1980’s, I heard a highly profiled professor making a comment after a lecture about a brand new technique, the polymerase chain reaction (PCR). His comment was full of doubt about this novel technology, and the message was something like: “it [PCR] can never become a widely used diagnostic tool due to the unavoidable contamination”. However, the PCR revolutionized life sciences from medicine to conservation genetics, the inventor was awarded with the Noble Prize, and many of us made careers using the very technique. Scientists, clinical diagnostics and forensic laboratories and others using PCR quickly learned to deal with contamination and build mechanisms to monitor for it. The contamination was there, but, it could be managed with the right laboratory environment, sample flow, and careful experimental design including proper sample handling and a set of controls.

For the past seven to eight years another new technology, second generation sequencing (SGS), also known as next generation sequencing (NGS) or massively parallel sequencing (MPS), is gaining more space in laboratories and scientific journals. This new wave of technologies already has had profound effects on human genomics, cancer biology, microbiology, ancient DNA studies and forensic genetics. Big data are fantastically interesting, will probably fundamentally change current views in biology, and are conceptualizing the basis of human diseases in a new way.

During the last couple of years the contamination worry has appeared again as it has with any molecular biology technique employing amplification. The studies under scrutiny surround the use of the NGS technology in the studies of e.g. modern viral [1] and ancient bacterial [2] pathogens, and whole genome sequence data of the domestic cow [3]*.*

In 2013, Xu *et al.* [1] published a study consisting of 92 seronegative, non-A – E, hepatitis patients from Chongqing, China. They used Solexa deep sequencing and found that all 10 sera pools had a 3.780 bp contig, which was located at the interface of *Parvoviridae* and *Circoviridae*. The authors designated the new virus provisionally as NIH-CQV. In the study, 63 of 90 patient samples (70%) were positive, but all those from 45 healthy controls were negative. The authors recommended further studies, but concluded that their “data indicate that a parvovirus-like virus is highly prevalent in a cohort of patients with non-A–E hepatitis.” Being so would have been of great medical importance, since non-A–E hepatitis is poorly understood and infected individuals have serious complications. Soon after, Naccache *et al.* [4], shed doubt on these findings. They discovered, using NGS, a highly divergent DNA virus, which also was at the interface between *Parvoviridae and Circoviridae*, and they tentatively called it a parvovirus-like hybrid virus (PHV). The authors detected the virus originally in various sets of clinical samples, and all strains were ~ 99% identical in nucleotide and amino acid sequences with each other and the NIH-CQV. Naccache *et al.* [4] then showed that the source of these viruses was contaminated commercial silica-binding spin-columns used in the sample preparation, and suggested that such contamination can be time dependent and geography specific. Smuts *et al.* [5] and Zhi *et al.* [6] also studied the silica-columns from the same company and confirmed the study by Naccache *et al.* [4], but they showed that silica-columns from some other companies were contamination free. Since silica in most commercial spin columns is derived from the cell walls of diatoms, the authors in Naccache *et al.* later postulated [7] that PHV/NIH-CQV could be a diatom virus, whereas Zhi *et al*. hypothesized that it originated from oomycetes [6].

Ancient DNA studies have always stressed rigid contamination control, and contextual interpretation of the results. Little consensus exists on sample collection and experimental study design of NGS-based studies which, may introduce another level of concern. Indeed, the study by Campana *et al.* [2] serves as an example. They tried to resolve the cause of *huey cocoliztli* (Great Pestilence in Nahautl), a hemorrhagic fever that killed almost half of the population in 1576 in Mexico. The authors used Helicos HeliScope and Illumina 2500 sequencing platforms for metagenomic sequencing to identify the pathogen in eight human remains from a known site of the *huey cocoliztli* outbreak from Spanish colonial times. They also took surrounding soil samples and four pre-colonial remains for comparative studies. Without the comparative sampling, the authors could have reported *Yersinia pestis* and *rickettsiosis* as causative pathogens, which now turned more likely to be false positive findings. Due to this observation, the authors suggested that target-enrichment methods should be used to confirm the presence of a pathogen.

Finally, mammalian genomes also have been studied for microbial contamination. Recently Merchant *et al.* [3] studied *Bos Taurus*, the domestic cow, whose genome was first assembled in 2009 from 35 million Sanger sequencing reads, and mapped into chromosomes. As common in such projects, small regions remained unmapped, and Merchant *et al*. [3] targeted those sequences. By use of Kraken system to classify the unmapped contigs, they surprisingly identified 173 small contigs that were of microbial origin. One of those was Bovine herpes virus 6, isolate Pennsylvania 47, which is a cattle-specific virus causing various diseases. This virus is a retrovirus, and the authors considered the possibility of viral insertion to the host genome, which they excluded during further investigation. The most common contaminants belonged to *Acinetobacter* (29 contigs), *Pseudomonas* (35 contigs) and *Stenotrophomonas* (27 contigs). Another unexpected contaminating contig of interest was 2.885 small contigs, earlier placed in chromosomes 1 to 10, which aligned to a human specific bacterium, *Neisseria gonorrhoeae*, strain TCDC-NG08107. Although this sequence is putatively a complete genome, it contained multiple sequences that seemed to derive from the cow and sheep genomes. These alarming findings caused GenBank temporarily to suppress the entry for this genome.

All these reports presented above suggest that when the scientific community is changing rapidly from Sanger sequencing to the next phase(s) in the sequencing technology, the importance of quality control and validation has to be emphasized. Microbial contamination is not yet fully understood, but not surprisingly it appears to be prevalent. There is a need for clear outline for detection and validation of new marker systems and setting thresholds for filtering out contamination in such studies as metagenomics [8]. Indeed, one such paper providing guidancce towards this direction has been published recently in *Investigative Genetics* [9].
